# Combination biologic therapy in bullous pemphigoid: two case reports and a focused review

**DOI:** 10.3389/fimmu.2026.1879665

**Published:** 2026-09-07

**Authors:** Aikaterini Kyriakou, Athanasia Tsaousi, Christos Minas, Maria-Emmanouela Anagnostaki, Antonios Fantakis, Parthena Meltzanidou, Anastasia Tsitlakidou, Athina-Ioanna Daponte, Elisavet Lazaridou, Aikaterini Patsatsi

**Affiliations:** 1 Center of Expertise on AIBD, 2nd Dermatology Department, Aristotle University School of Medicine, Papageorgiou General Hospital, Thessaloniki, Greece; 2 2nd Dermatology Department, Aristotle University of Thessaloniki, Thessaloniki, Greece; 3 Research Collaborator, Aristotle University of Thessaloniki, Thessaloniki, Greece; 4 1st Surgical Department, Aristotle University of Thessaloniki, Thessaloniki, Greece

**Keywords:** autoimmune disease, biologic therapy, blistering disorder, bullous disease, bullous pemphigoid, targeted therapy

## Abstract

Bullous Pemphigoid (BP) is the most common autoimmune blistering disorder, predominantly affecting elderly patients and often requiring prolonged immunosuppression. Conventional therapies are frequently limited by adverse effects and incomplete disease control, particularly in refractory cases. Increasing understanding of BP pathogenesis has supported the use of targeted biologic therapies; however, evidence regarding combination approaches remains limited. We report two cases of refractory BP treated with combination biologic therapy and provide a focused review of the literature. In the first case, partial response to omalizumab was followed by complete disease control after the addition of dupilumab, allowing successful corticosteroid discontinuation. In the second case, a patient with concomitant psoriasis achieved marked improvement with dupilumab in combination with ongoing IL-17 inhibition (secukinumab), resulting in effective control of both BP and psoriasis. These observations highlight the benefit of simultaneous targeting of complementary inflammatory pathways, including IgE-mediated, Th2, and Th17 axes. Combination biologic therapy may represent a promising strategy in refractory BP, particularly in patients with complex immunologic profiles or coexisting inflammatory diseases.

## Introduction

Bullous Pemphigoid (BP) is the most common autoimmune subepidermal blistering disorder, predominantly affecting older adults and characterized by autoantibodies directed against the hemidesmosomal proteins BP180 and BP230 (1, 2). Clinically, BP presents with a broad spectrum of manifestations, ranging from nonbullous pruritic and urticarial lesions to widespread tense blistering, reflecting significant underlying immunologic heterogeneity (1, 2).

Conventional management relies on systemic corticosteroids, either as monotherapy or in combination with immunosuppressive agents such as azathioprine or mycophenolate mofetil (1). While often effective, these treatments are associated with considerable adverse effects, particularly in elderly patients with multiple comorbidities. Current guideline recommendations therefore emphasize minimizing corticosteroid exposure and prioritizing safer, steroid-sparing approaches whenever feasible (1).

Advances in the understanding of BP pathogenesis have revealed a complex interplay between autoreactive B cells, IgG and IgE autoantibodies, complement activation, and type 2 (Th2)-skewed inflammation (2, 3). Elevated IgE levels and eosinophilia are frequently observed, and IgE autoantibodies targeting BP180 contribute directly to disease activity through mast cell activation and eosinophil recruitment (2, 3). These insights have led to the increasing use of targeted biologic therapies, including B-cell depletion (rituximab), IgE blockade (omalizumab), and IL-4/IL-13 pathway inhibition (dupilumab).

Emerging evidence supports the efficacy of these agents as monotherapy, with cohort studies reinforcing the role of dupilumab and systematic reviews confirming the benefit of multiple biologic classes in BP (4, 5). More recently, attention has shifted toward combination biologic therapy, particularly in patients with refractory disease or overlapping immunologic phenotypes. Reports describing combinations such as dupilumab plus omalizumab and rituximab-based regimens have demonstrated encouraging results in terms of disease control and steroid sparing (6–13).

Despite these advances, the use of combination biologic therapy remains largely empirical. There is limited consensus regarding optimal patient selection, therapeutic combinations, and the role of disease phenotype and comorbidity burden in guiding treatment decisions.

In this context, the present study aims to appraise combination biologic therapy in BP through a phenotype- and comorbidity-oriented framework, integrating current evidence with the presentation of two illustrative cases.

## Case presentations

### Case 1

A 68-year-old woman with a history of refractory BP presented with persistent disease activity characterized by recurrent exacerbations and incomplete response to prior therapies. The diagnosis had been previously established based on clinical presentation and supportive immunopathological findings (**Table 1**).

**Table 1:** Baseline clinical and immunological characteristics of the two patients.

Parameter	Case 1	Case 2
Age (years)	68	47
Sex	Female	Female
BPDAI score at baseline	32	27
Eosinophil count (×10³/μL)	0.14	0.01
Total IgE (IU/mL)	240.0	90.8
Anti-BP180 antibodies (RU/mL)	46.8	65.5
Anti-BP230 antibodies (RU/mL)	15.1 (negative)	14.1 (negative)

Initial management included systemic corticosteroids in combination with dapsone; however, this regimen failed to achieve adequate disease control. Consequently, dapsone was discontinued, and therapy with omalizumab was initiated alongside ongoing corticosteroid treatment. Although this approach resulted in partial clinical improvement, persistent disease activity precluded corticosteroid tapering. Therefore, dupilumab was added, while omalizumab was continued, establishing a dual biologic regimen based on the complementary mechanisms of action of the two biologic agents, with the aim of achieving complete disease control and facilitating corticosteroid withdrawal.

Following initiation of combination therapy, the patient demonstrated progressive clinical improvement, with complete disease control achieved within six months. At the 6-month follow-up visit, the BPDAI score had decreased from 32 to 0. Systemic corticosteroids were gradually tapered and successfully discontinued without relapse. No significant adverse events related to combination biologic therapy were observed during the 6-month follow-up period.

This case highlights the efficacy of concurrent dual-pathway inhibition in BP, following stepwise therapeutic escalation from conventional therapy to combined IgE and Th2 blockade. The observed response underscores the potential of combination biologic therapy in patients with refractory disease and incomplete response to single-agent approaches.

### Case 2

A 47-year-old woman with refractory BP presented with recurrent disease flares despite prior treatment. Her therapeutic history included systemic corticosteroids, rituximab, and omalizumab, all of which resulted in suboptimal disease control.

The patient had a personal history of psoriasis and psoriatic arthritis, both previously in remission. During the course of refractory BP, psoriasis and psoriatic arthritis flared, prompting the initiation of secukinumab for the management of the psoriatic disease. As BP remained active despite previous treatment with systemic corticosteroids, rituximab, and omalizumab, dupilumab was added shortly thereafter while secukinumab was continued, to achieve better control of BP while preserving the clinical response of the psoriatic disease.

This approach resulted in significant clinical improvement, marked reduction in disease activity, and substantial enhancement in quality of life. At the 6-month follow-up visit, the BPDAI score had decreased from 27 to 0. Complete tapering and discontinuation of corticosteroids were achieved, with resolution of steroid-related adverse effects, including impaired glycemic control, fatigue, and muscle weakness. No adverse events related to combination biologic therapy were observed during the 6-month follow-up period.

This case highlights the feasibility and efficacy of dual-pathway targeting, with simultaneous modulation of Th2 and Th17 axes, allowing effective control of both BP and psoriasis.

## Discussion

The therapeutic landscape of BP is undergoing a progressive shift from broad immunosuppression toward targeted, mechanism-based interventions (1–3). This evolution reflects a more nuanced understanding of BP pathogenesis and is particularly relevant in the elderly population, in whom treatment-related morbidity is a major concern (1, 2).

Systemic corticosteroids, while effective, are associated with significant adverse outcomes when used long term, prompting guideline recommendations to minimize their use and explore safer alternatives (1). Biologic therapies have emerged as promising candidates in this regard, offering targeted modulation of specific immune pathways involved in disease pathogenesis (**Table 2**).

**Table 2:** Previously reported cases of combination biologic therapy in bullous pemphigoid.

Published paper	n	Age (yr)/ Sex	Phenotype/ Clinical scenario	Comorbidities/ Trigger	Combination regimen (pathways targeted)	Failed prior therapies	Duration of combination	Outcome	Adverse events
A — Omalizumab + Dupilumab (anti-IgE + anti–IL-4Rα) | Refractory/Type 2–driven BP									
Seyed Jafari et al., 2021 (8) Front Immunol	1	70/M	Refractory BP with persistent disease despite immunosuppression	Metabolic syndrome, T2DM, obesity, hypertension	OMZ 300 mg q4w + Dupilumab 600 mg → 300 mg q2w + MMF Anti-IgE + anti–IL-4Rα	Topical CS, dapsone (150 mg/day), MTX, MMF (9 months, partial response)	~10 months	Complete remission; CS discontinued; anti-BP180 ↓ 43→25 U/mL	None
Boisseau et al., 2025 (7) Acta Derm Venereol	6	67–89 4M/2F	Refractory BP; two cases of ICI-associated BP	Active cancer (n=3), CKD (n=1), pulmonary TB sequelae (n=1)	OMZ 300 mg q4w + Dupilumab 600 mg → 300 mg q2w (add-on to topical CS ± doxycycline) Anti-IgE + anti–IL-4Rα	Topical CS, doxycycline; immunosuppressants contraindicated in 5/6	Median 49 weeks (range 18–140)	Lesion regression in all patients; 2 relapses after discontinuation, both responsive to reintroduction	No biologic-related adverse events; 1 unrelated death (SCC recurrence/COVID-19)
Ratnarajah et al., 2025 (10) JAAD Case Rep	2	54/M 59/M	Severe refractory BP	Pt 1: psoriasis, recent aortic dissection surgery Pt 2: T2DM, ESRD on dialysis, renal transplant, active prostate cancer	OMZ 300 mg q2–4w + Dupilumab 300 mg q2w + MMF Anti-IgE + anti–IL-4Rα	Pt 1: topical CS, MTX Pt 2: prednisone, doxycycline, OMZ (partial); IVIg contraindicated	10 months (Pt 1); two 6-month rescue courses (Pt 2)	Disease clearance in both patients; successful CS tapering; remission >18 mo (Pt 1) and >12 mo (Pt 2)	None; both deaths unrelated to therapy
Victory et al., 2025 (9) JAAD Case Rep	1	43/F	Immune checkpoint inhibitor–associated BP	Hodgkin lymphoma treated with nivolumab; hypothyroidism, obesity, epilepsy	OMZ 300 mg q4w (initiated first) → Dupilumab 300 mg q2w added Anti-IgE + anti–IL-4Rα	Prednisolone, doxycycline, clobetasol, MMF (→ cytopenia), IVIg, rituximab, plasma exchange (×6 cycles)	~6 months combination; dupilumab monotherapy thereafter	Remission on dupilumab monotherapy at 2-year follow-up; anti-BP180 ↓ 193→81 U/mL	None attributable to biologics
B — Dupilumab + Secukinumab (anti–IL-4Rα + anti–IL-17A) | BP with concomitant psoriasis									
Cao et al., 2025 (15) Indian J Dermatol Venereol Leprol	1	75/M	BP with coexisting psoriasis	Coronary artery disease, hypertension	Dupilumab 600 mg → 300 mg q2w + Secukinumab 300 mg weekly Anti–IL-4Rα + anti–IL-17A	Apremilast (ineffective)	5 months follow-up	Near-complete resolution within 6 weeks; sustained control of BP and psoriasis at 5 months	None
C — Rituximab + Omalizumab (anti-CD20 + anti-IgE) | Autoantibody-driven/severe refractory BP									
Le et al., 2024 (11) JAMA Dermatol	NR*	NR	Refractory BP	NR	Rituximab + OMZ Anti-CD20 + anti-IgE	NR	NR	Clinical improvement reported	NR
Kwon et al., 2023 (13) J Dermatol	1	NR	Refractory BP with insufficient response to rituximab	NR	OMZ added to ongoing rituximab Anti-CD20 + anti-IgE	Rituximab alone (insufficient)	NR	Improved disease control after addition of OMZ	NR
D — Other dual biologic therapy in patients with BP and concomitant immune-mediated disease									
Gisondi et al., 2023 (6) Dermatol Ther (Heidelb)	1	70/F	BP with concomitant psoriasis	Psoriasis	Dupilumab (for BP) + Guselkumab (for psoriasis) Anti–IL-4Rα + anti–IL-23	NR	6 months	No disease flare; treatment well tolerated	None

BP, bullous pemphigoid; CAD, coronary artery disease; CKD, chronic kidney disease; CS, corticosteroids; ESRD, end-stage renal disease; ICI, immune checkpoint inhibitor; IVIg, intravenous immunoglobulin; MMF, mycophenolate mofetil; MTX, methotrexate; NR, not reported; OMZ, omalizumab; q2w/q4w, every 2/4 weeks; SCC, squamous cell carcinoma; T2DM, type 2 diabetes mellitus; TB, tuberculosis; wk, week. *Le et al., 2024 reported a case series; individual patient-level data were not available for tabulation.

The rationale for biologic therapy is supported by the recognition that BP involves not only autoantibody-mediated tissue injury but also a broader inflammatory network characterized by Th2 polarization, eosinophilic activation, and IgE-mediated immune responses (2, 3). Within this framework, combination biologic therapy represents a logical extension of single-agent treatment, particularly in patients with complex or refractory disease.

The combination of dupilumab and omalizumab is among the most frequently reported strategies, demonstrating improvement in pruritus, blistering, and corticosteroid dependence (7–9, 12). Mechanistically, this approach allows simultaneous inhibition of IL-4/IL-13 signaling and IgE-mediated pathways, resulting in broader suppression of type 2 inflammation (2, 3). The combination of dupilumab and omalizumab, as illustrated in the present cases, further supports the concept of complementary inhibition of IgE-mediated and Th2-driven inflammatory pathways.

The growing evidence supporting dupilumab monotherapy further strengthens its role as a therapeutic backbone in BP, with recent cohort data demonstrating favorable efficacy and safety outcomes (4). Combination regimens may therefore be particularly useful in patients with partial responses or persistent disease activity.

Similarly, combinations involving rituximab may be especially relevant in severe or autoantibody-driven BP. By targeting B-cell-mediated autoantibody production, rituximab addresses a central pathogenic mechanism, while adjunctive agents such as omalizumab may provide additional control of IgE-mediated inflammation (5, 11, 13).

An important emerging concept is the role of clinical and immunological phenotyping in guiding treatment selection. Patients with elevated IgE levels, eosinophilia, and severe pruritus may preferentially benefit from Th2-targeted therapies, whereas those with high anti-BP180 IgG titers and extensive blistering may respond better to B-cell-directed approaches (4, 5, 7, 12).

Equally important is the role of comorbidities. Because BP frequently occurs in patients with neurologic, cardiovascular, metabolic, or oncologic comorbidities, treatment tolerability often determines therapeutic feasibility as much as efficacy (1, 2). Biologic agents such as dupilumab and omalizumab offer favorable safety profiles and facilitate corticosteroid sparing (4, 6, 7, 12).

An additional layer of complexity arises in patients with coexisting immune-mediated diseases, most notably psoriasis. In such cases, therapeutic strategies may need to address divergent inflammatory pathways, including Th2-driven mechanisms implicated in BP and Th17/IL-23 axis activation characteristic of psoriasis. Recent reports have described the successful use of combination regimens incorporating dupilumab with psoriasis-directed biologics, such as IL-17 inhibitors (e.g., secukinumab), allowing simultaneous control of both conditions (14, 15). These observations highlight the potential utility of dual-pathway inhibition, in which Th2 and Th17 axes are targeted concurrently.

Our second case further illustrates the complexity of managing BP in patients with concomitant immune-mediated diseases. Although the improvement in BP was likely primarily attributable to dupilumab, secukinumab was continued throughout treatment and may also have contributed to disease control. The individual contribution of each biologic cannot be determined from a single case. Nevertheless, emerging evidence suggests that inflammatory pathways beyond classical Th2 immunity, including the IL-17 axis, may participate in BP pathogenesis through the recruitment and activation of neutrophils and amplification of the inflammatory cascade (2, 3). Therefore, while the principal therapeutic effect on BP was likely mediated by dupilumab, a contributory role of ongoing IL-17 blockade cannot be excluded.

From a mechanistic standpoint, this approach is particularly compelling, as emerging evidence suggests that BP may, in certain contexts, involve overlapping inflammatory pathways beyond classical Th2 polarization (2, 3). Similarly, in immune checkpoint inhibitor-associated BP, where immune dysregulation is multifactorial, combination biologic therapy has demonstrated clinical benefit and may represent a rational therapeutic strategy (9).

Taken together, these findings underscore the importance of individualized, comorbidity-driven treatment selection, in which therapeutic decisions are guided not only by disease severity but also by the presence of coexisting inflammatory conditions and patient-specific risk factors.

Despite these promising developments, the current evidence base remains limited. Most available data is derived from case reports, small case series, and retrospective analyses, with considerable heterogeneity in patient selection, treatment regimens, concomitant therapies, follow-up duration, and outcome measures (5, 7, 8, 10, 12). Consequently, direct comparisons between studies are challenging, and the generalizability of reported outcomes remains uncertain.

Furthermore, publication bias represents an important limitation, as successful and unusual cases are more likely to be reported than treatment failures or negative experiences. This may result in an overestimation of the efficacy and safety of combination biologic therapy in routine clinical practice. The absence of prospective controlled studies and randomized clinical trials further limits the ability to determine whether combination biologic therapy is superior to optimized monotherapy, sequential biologic use, or conventional immunosuppressive approaches. Larger multicenter studies and prospective registries will be essential to better define patient selection criteria, evaluate long-term safety, and establish evidence-based treatment algorithms.

In conclusion, combination biologic therapy represents a promising and evolving strategy in BP. A mechanism-based approach to combination biologic therapy, tailored to the individual patient’s clinical characteristics and treatment response, may optimize therapeutic outcomes and support the transition toward precision medicine in this complex disease.
